# A healthy live birth after mosaic blastocyst transfer in preimplantation genetic testing for GATA1-related cytopenia combined with HLA matching

**DOI:** 10.1186/s12920-024-01951-2

**Published:** 2024-07-03

**Authors:** Huiling Xu, Jiajie Pu, Zhengzhong Wu, Yulong Huang, Chanlin Han, Xuemei Li

**Affiliations:** 1 Department of Reproductive Medicine, Affiliated Shenzhen Maternity & Child Healthcare Hospital, Southern Medical University (Shenzhen Maternity & Child Healthcare Hospital), Shenzhen, Guangdong China; 2 Department of Bioinformatics, 01life Institute, Shenzhen, 518000 Guangdong China

**Keywords:** PGT-M, HLA typing, *GATA1*, Mosaic blastocyst transfer

## Abstract

**Background:**

GATA1-related cytopenia (GRC) is characterized by thrombocytopaenia and/or anaemia ranging from mild to severe. Haematopoietic stem cell transplantation (HSCT) is a healing therapeutic choice for GRC patients. We identified a novel pathogenic variant (*GATA1*: c.1019delG) in a boy with GATA1-related cytopenia. Then we performed preimplantation genetic testing (PGT) in this GRC family. After a mosaic embryo transfered, a healthy and HLA-compatible with the proband baby was delivered.

**Case presentation:**

The proband is a 6-year-old boy who was diagnosed to have transfusion-dependent anaemia since 3 year old. Whole-exome sequencing (WES) showed that the proband has a hemizygous variant c.1019delG in *GATA1*, which is inherited from his mother. His parents decided to undergo PGT to have a health and HLA-compatible offspring. After whole genome amplification (WGA) of biopsied trophectoderm (TE) cells, next generation sequencing (NGS)-based PGT was preformed to analyse embryos on chromosomal aneuploidy, target mutation and HLA typing. There were 3 embryos HLA-matched to the proband. The genotypes of the 3 embryos were heterozygous variant, hemizygous variant, normal respectively. After a heterozygous, mosaic partial trisomy (chr)16, and HLA-matched embryo transfer, a healthy baby was delivered and whose HSCT is compatible with the proband.

**Conclusions:**

NGS-based PGT-HLA is a valuable procedure for the treatment of GATA1-related cytopenia caused by *GATA1* variants, or other haematological disorders, oncological and immunological diseases. Furthermore, our study reconfirms that mosaic embryos transfer would bring healthy offspring.

## Background

GATA-BINDING PROTEIN 1 (GATA 1, OMIM: * 305371), an important haematopoietic transcription factor, encodes a zinc finger DNA-binding transcription factor that plays a critical role in differentiation and maturation of erythroid and megakaryocytic cell lines [1]. When germline mutation occurs in *GATA1*, it causes a variety of X-linked recessive forms of hereditary thrombocytopaenia and dyserythropoietic anaemia [2]. *GATA1*-related cytopenia (GRC) is characterized by thrombocytopaenia and/or anaemia ranging from mild to severe. According to different phenotypes, GRC could be classified as various diseases including X-linked thrombocytopaenia (XLT), X-linked thrombocytopaenia with thalassaemia (XLTT), congenital erythropoietic porphyria (CEP), transient myeloproliferative disorder (TMD) and acute megakaryoblastic leukaemia (AMKL) associated with trisomy 21 [3]. Haematopoietic stem cell transplantation (HSCT) is a healing therapeutic choice for affected patients with *GATA1* variant if HLA-matched donors are available [4]. Preimplantation genetic testing-human leukocyte antigen (PGT-HLA) refers to HLA typing of single or few cells biopsied from in vitro fertilized preimplantation embryos, which not only identifies unaffected embryos but also characterizes the embryos that are HLA compatible with an already affected child who requires HSCT. Since the inheritance pattern of GRC is X-linked recessive, females who carry one copy of the pathogenic *GATA1* gene on one of their X chromosomes are generally unaffected by the disorder as they carry one copy of the normal gene on their other X chromosome. Despite that, the mutated gene can be passed on to their children. In case where a male baby who inherits the mutated gene from his mother, the boy would develop the disorder since he only has one X chromosome; he does not have an additional copy of the unaffected *GATA1* gene to compensate. Therefore, GRC is more common in males than in females [5].

In this study, we identified a novel frameshift variant (c.1019delG) in the *GATA1* gene causing GRC in a proband who inherited the variant from his unaffected mother. PGT-HLA was performed to select the HLA-compatible and GRC-free embryo. Finally, a healthy baby was delivered after the mosaic embryo transfer.

## Case presentation

### Patients

The proband is a 6-year-old boy, who was initially diagnosed to have mild anaemia during a routine health check at the age of 6 months. Subsequently, his condition has progressively deteriorated over time. By the age of three, his condition had escalated into severe anaemia with a haemoglobin level of 4.7 g/dl, necessitating monthly blood transfusions to manage the situation. The proband underwent bone marrow aspirations on two separate occasions at different hospitals, both of which indicated no abnormalities. However, the high performance liquid chromatography (HPLC) chromatogram revealed an elevated level of HbF (21.8%), with HbA constituting 76.5% and HbA2 at 1.7%. Notably, the proband's HbF level is approximately 20-fold higher than the reference range. The haemogram and HPLC results for the proband and his partents are presented in Table 1. The proband is the firstborn child of the couple, and since his birth, they have experienced two miscarriages during early embryonic stages, without any genetic testing performed on the miscarriage tissue. The karyotype analysis conducted on both parents revealed no abnormalities. Neither parent nor any other family member has anaemia or a history of blood disorders. Given the significantly high HbF level in the proband, we initially hypothesized thalassaemia as a potential cause. We performed polymerase chain reaction (PCR) to detect possible pathogenic variants in the alpha- and beta-globin genes within the proband's genome; however, no such variants were detected. To identify the underlying causative variants, whole exome sequencing (WES) was carried out on the proband's sample. Suspected variants identified by WES were validated by Sanger sequencing. Informed consent was obtained from relevant members to participate in this study.

**Table 1:** Haematological data of proband and parents

Individual	Hgb (g/dl)	MCV (fl)	HbF (%)	HbA (%)	HbA_2_ (%)
Normal range	Femal:11.0–15.0 Male:12.0–16.0	80.0–100.0	< 2.5	96.5–97.5	< 3.5
Proband	4.6	89.8	21.8	76.5	1.7
Mother	13.6	96.7	1.1	96.3	2.6
Father	13.6	86.9	–	97.5	2.5

Normal values are shown at top. *Hgb* hemoglobin concentration, *MCV* mean corpuscular volume, *HbF* percentage of foetal hemoglobin, *HBA* percentage of adult hemoglobin, *HbA*_*2*_ percentage of hemoglobin A2

**Fig. 1:**
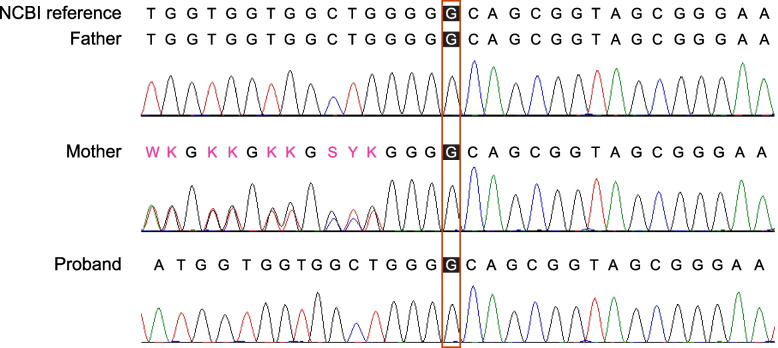
Sanger sequencing results of the *GATA1* variant c.1019delG. The proband is hemizygous for the variant. The mother was found to be heterozygous for the variant while the father was normal. K: keto (G or T); S: strong (G or C); W: weak (A or T); Y: pyrimidine (T or C)

The WES showed that the proband has a hemizygous frameshift variant of c.1019delG in the *GATA1* gene, which was confirmed by Sanger sequencing. The maternal variant is heterozygous while the father is normal (Fig. 1). The identified variant has not been documented in any of the commonly referenced population databases as of 15th January 2022, including gnomAD, ClinVar, and HGMD or any literature. According to the American College of Medical Genetics and Genomics and the Association for Molecular Pathology (ACMG/AMP) guidelines [6], the novel variant is categorized as likely pathogenic (PVS1 + PM2).

### Ovarian stimulation, fertilization, embryo culture, TE biopsy

The patient was stimulated using a progestin-primed ovarian stimulation (PPOS) protocol. Oocyte retrieval was conducted 36 h subsequent to the administration of human chorionic gonadotropin (hCG) trigger. Twenty oocytes were successfully harvested and inseminated by ICSI. Fifteen of these oocytes were fertilized normally as indicated by the presence of two pronuclei. Embryos were cultured with sequential media (G1, G2, Vitrolife, Sweden) under a temperature of 37 °C and gas concentrations of 5% O2, 6% CO2, and 89% N2. A total of six viable blastocysts were obtained. All of these blastocysts underwent trophectoderm (TE) biopsy on day 6 via laser. Approximately 5–10 cells were extracted from the TE layer of each blastocyst. The biopsied cells were rinsed three times using 1 × PBS solution without Mg2 + and Ca2 + , and then stored in PCR tubes pre-filled with 5μL of cell lysis buffer, which is ready for subsequent whole-genome amplification (WGA). Biopsied embryos were cryopreserved with vitrification method (VT101, Kitazato, Janpan).

### Preimplantation genetic testing of the embryos

Whole genome amplification (WGA) was performed on the biopsied cells following the protocol of multiple annealing and looping-based amplification cycles (MALBAC) (Yikon Genomics Inc, China). Then WGA product of each embryo was subjected to Sanger sequencing for direct identification of the variant site (*GATA1*: c.1019delG). As only a limited number of cells could be used for amplification; it is hard to avoid allele drop-out (ADO). To prevent misdiagnosis, haplotyping was conducted using single nucleotide polymorphism (SNP) markers with a sequencing depth ≥ 100 × within the 1 Mb genomic region flanking the targeted gene through targeted capture sequencing. SNP markers that displayed homozygosity in the father and heterozygosity in the mother were selected as informative SNP markers for the haplotype linkage analysis. The proband haplotypes were used as the reference to determine if the embryos carrying the parental chromosome that harbouring the variant. Further details on these methods can be found in previous studies [7, 8].

Besides the detection of *GATA1* variant, copy number variation (CNV) analysis was also carried out on all embryos to prevent embryonic abortion, death or other problems may be caused by embryonic chromosomal abnormalities. Any deletion or duplication larger than 4 Mb and mosaicism more than 30% within the embryo will be reported.

Five HLA regions (HLA-*A*, HLA-*B*, HLA-*C*, HLA-*DR* and HLA-*DQ*) were detected to ensure HLA of embryo is compatible with the proband. To avoid misdiagnosing caused by HLA recombination, haplotyping of the chromosome 6 of the embryos were meticulously analyzed for all SNP markers within 2 Mb upstream and downstream of the HLA gene, with comparison to the results from the mother, father, affected child. Detailed method is thoroughly described as previous reports [9, 10]. The order of priority for embryo transfer depending on the embryo quality and the PGT results. Clinical pregnancy is defined as the presence of a foetal heartbeat by sonography 28 days after frozen embryo transfer (FET). Amniotic fluid at 17 weeks of gestation was collected for chromosomal microarray analysis (CMA), HLA haplotyping and mutation analysis on the *GATA1* gene to validate the diagnosis of PGT.

**Fig. 2:**
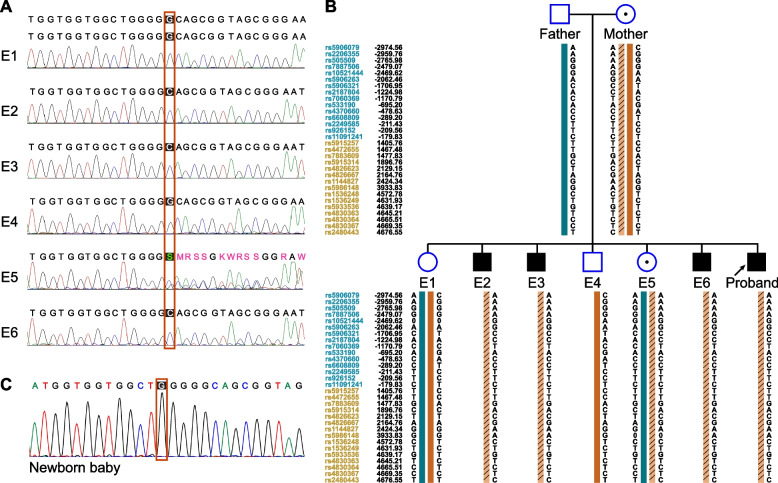
The PGT-M results of *GATA1*: c.1019delG. **A** Sanger sequencing result of the 6 blastocysts. K: keto (G or T); M: amino (A or C); R: purine (G or A); S: strong (G or C); W: weak (A or T). **B** Schematic diagram representing the SNP-based haplotype analysis of the family members and embryos of *GATA1*. Among the 6 embryos, one blastocyst carried a heterozygous variant, three carried hemizygous variant, while the rest two were unaffected. The SNP ID numbers highlighted in dark blue and orange refer to the upstream and downstream informative SNPs, respectively. The dark blue and the dark orange bars represent the normal haplotype of the father and the mother, respectively. The slashes filled orange bar denotes the variant haplotype of the mother. A 0/0 in the haplotype means unsuccessful genotyping for the marker in that sample.** C** Prenatal diagnosis of amniotic fluid DNA. Sanger sequencing showed that the newborn baby was unaffected for *GATA1* gene

**Fig. 3:**
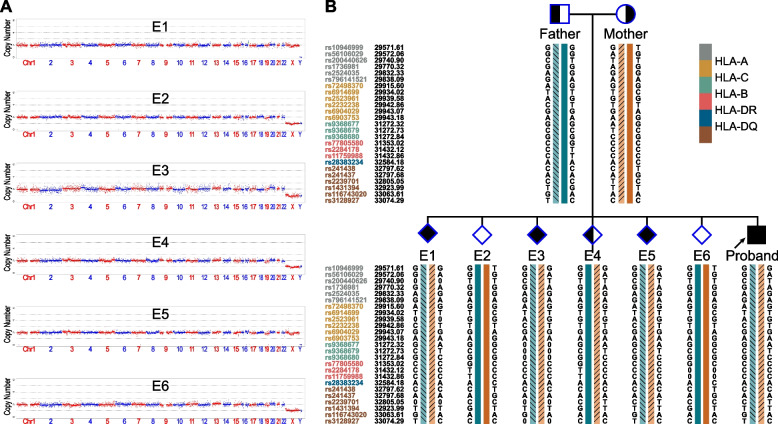
CNV analyses and SNP-based haplotype analyses results of the family members and embryos of HLA. **A** CNV analyses results of the embryos. **B** The SNP-based haplotype linkage analyses of HLA. All the embryos were identified with chromosomal normality except embryo E1 which is mosaic partial trisomy (chr) 16. And there were 3 embryos HLA-matched with the proband. The dark blue and the dark orange bars represent the proband-HLA-unmatched haplotype of the father and the mother, respectively. The slashes filled orange bar denotes the proband-HLA-matched haplotype of the mother, and the backslashes filled light blue bar denotes the proband-HLA-matched haplotype of the father. Color keys of the upstream and downstream informative SNPs of the five HLA gene regions (HLA-*A*, HLA-*B*, HLA-*C*, HLA-*DR* and HLA-*DQ*) were showed in the figure legend on the right. A 0/0 in the haplotype means unsuccessful genotyping for the marker in that sample

Sanger sequencing showed that one blastocyst carried a heterozygous variant, 3 carried hemizygous variant, and the remaining two were unaffected (Fig. 2A). Those results were consistent with haplotype linkage analysis (Fig. 2B). All the embryos were identified as chromosomally normal except E1 embryo which is mosaic partial trisomy (chr)16 (Fig. 3A). Three embryos were HLA-matched with the proband (Fig. 3B). PGT outcomes of biopsied blastocysts are summarized in Table 2.

**Table 2:** Detection result summary of biopsied blastocysts

Embryo number	Grading^a^	*GATA1*	Aneuploidy	HLA
E1	5AB	Normal	46, XN, + 16q (q23.1 → q24.1, ~ 11 Mb, × 3, mos, ~ 30%)	Matched
E2	6BB	c.1019delG hemizygous	46, XN	Not Matched
E3	5BB	c.1019delG hemizygous	46, XN	Matched
E4	5BB	Normal	46, XN	Not Matched
E5	6CB	c.1019delG heterozygous	46, XN	Matched
E6	3CB	c.1019delG hemizygous	46, XN	Not Matched

^a^The embryo quality was assessed following the Gardner grading system [11]

### Prenatal diagnosis and pregnant outcome

The first embryo transfer was the heterozygous of *GATA1*:c.1019delG, HLA-matched, diploid embryo E5, but this frozen embryo transfer cycle was failed. After detailed genetic counseling, the couple decided to transfer embryo E1 which was the *GATA1*-variant-free, mosaic partial trisomy (chr)16, and HLA-matched. Finally, clinical pregnancy was achieved after embryo transfer. Prenatal diagnosis at 17 weeks of gestation showed that the foetus was free of the *GATA1* gene variant and euploidy, as well as HLA-matched with the proband (Fig. 2C). At last, a healthy male baby was delivered.

## Discussion and conclusions

GATA1, the first member of the GATA transcription factor family discovered by Evan in 1988 [12], is located at chromosome Xp11.23. *GATA1* variant could cause variable degrees and kinds of abnormalities of the haematological system scuh as leukaemia, anaemia, thrombocytopaenia [13–15]. *GATA1* defects are phenotypically heterogeneous even for different substitutions at the same amino acid position [16]. But the mechanism of human haematopoietic diseases caused by *GATA1* dysfunction remain to be further clarified [17]. *GATA1* defect is an X-linked recessive genetic disorder. But there are some female carriers reported to have a milder phenotype, composed of mild anaemia and thrombocytopaenia which relates to the proportion of cells containing the mutant *GATA1* allele on the active X chromosome [18]. In our case, the morther had normal haematological parameters and did not show unbalanced X chromosome inactivation. *GATA1* is critical for transitioning haemoglobin from HbF to HbA and HbA_2_. Elevated HbF may be observed in patients with *GATA1* variant. The proband suffered from severe anaemia with an Hb of 4.6 g/dL and a high HbF level of 21.8%. The phenotypes are consistent with haematological disorder caused by the *GATA1* variant. The pathogenic variant c.1019delG found in the proband is a frameshift variant which might damage the protein function because of premature stop codon leading to probably nonsense-mediated mRNA decay (NMD) of the *GATA1* mRNA. Bioinformatic analysis suggested the variant to be pathogenic. The variant hasn’t been reported previously, and we provided here the first description of a new frameshift variant in the *GATA1* gene (c.1019del, p.Gly340Alafs*14) causing GRC.

Since Verlinsky et al. first successfully applied PGT-M combined with HLA in a Fanconi anaemia family in 2001 [19], PGT-M with HLA typing for couples with children affected by genetic disorders that require HLA-identical stem cell transplantation therapy has a growing number of reports [20–22]. The European Society for Human Reproduction and Embryology (ESHRE) PGT-M Working Group advises that PGT-HLA protocol must include a minimum of one fully informative marker located at each of the following region: telomeric to the HLA-*A*, between HLA-*A* and HLA-*B*, between HLA-*B* and HLA-*DRA*, between HLA-*DRA* and HLA-*DQB1* and downstream to HLA-*DQB1* [23]. In this study, we chose NGS-based SNPs as informative markers. There were 27, 29, 21, 29, 29, 30 available SNP markers in HLA typing analysis for embryos E1 to E6 (data not shown), respectively. Such abundant SNP makers leads to a personalized diagnosis and accurate recognition of HLA recombination. The haematopoietic stem cells from umbilical cord blood of the HLA-matched newborn were collected and used for transplantation to, and cure of, the proband.

After implantation failure of E5 embryo, there was left only with one HLA-matched, unaffected, but mosaic embryo available. The couple received detailed genetic counseling and then decided to transfer this mosaic embryo. Fortunately, the woman became pregnant, and prenatal diagnosis did not reveal any detectable genetic abnormalities. This resulted in the delivery of a healthy male baby, who showed no apparent phenotypic anomalies. Embryonic mosaicism, defined as the presence of two or more genetically different cell lineages in an embryo, mostly originates from mitotic errors during the post-zygotic stage [24]. At the blastocyst stage, the incidence of mosaicism estimated using NGS methods has been reported ranging from 6.6% to 29.1% [25–27]. Some factors, such as slow developing, poor-quality blastocysts, semen quality, paternal age might increase the incidence of mosaicism. Different biopsy protocols may also have an impact on the mosaic blastocyst rate [28–31]. Since the first successful pregnancies after transfer of mosaic embryos reported by Greco et al. [32], the scientific community has aroused great interest in the clinical outcome of mosaic embryo transfer. Although there are some retrospective studies and meta-analysis have reported the capability of mosaic embryo transfer leads to healthy lives births, transferred mosaic embryo have significantly reduced implantation rates, lower live birth rate, as well as higher rate of spontaneous abortion compared with the euploid group [33–35]. The possible explanation that a healthy live birth was given after the transfer of mosaic embryo E1 is that chromosomally abnormal cells will be expelled from blastocysts as arrested cells/cellular debris during embryo development, a process called embryos' self-correction [36, 37]. Besides, the limited number of biopsied TE cells, which originate from extraembryonic lineage, may not be representative of the whole embryo or even the whole TE itself [38]. Although many reports of mosaic embryo transfer have shown apparently healthy live births, a few cases regarding the risk of live birth caused by the genetic abnormality have also been reported [39–42]. Combining conclusions from those studies suggested that we should take an optimistic, but cautious attitude towards the mosaic embryo transfer.

In conclusion, we identified a novel pathogenic *GATA1* variant (c.1019delG) in a boy with *GATA1*-related cytopenia. NGS-based PGT-A, PGT-M and PGT-HLA were performed. After an embryo implantation failure, an HLA-matched, unaffected, but mosaic embryo was transferred, and fortunately, allowing the birth of healthy baby who was also HLA-identical to the affected sibling. Here we report a case of successful HSCT from siblings created by embryo selection through PGT-M combined with PGT-HLA. It is a valuable procedure for the treatment of children with some haematological, oncological, or immunological diseases. Furthermore, our study reconfirms that mosaic embryo transfer would bring healthy offspring, which added evidence on the preferred outcome of mosaic embryo transfer. But more investigations and consensus are needed for guiding the transfer of mosaic embryo with a promising pregnancy outcome.

## Data Availability

The datasets generated and analyzed during the current study are available in the Chinese GSA (https://ngdc.cncb.ac.cn/gsa-human/) repository, accession number HRA007749.

## Abbreviations

GRC: GATA1-related cytopenia
HSCT: Haematopoietic stem cell transplantation
PGT: Preimplantation genetic testing
WES: Whole-exome sequencing
TE: Trophectoderm
NGS: Next generation sequencing
XLT: X-linked thrombocytopaenia
XLTT: X-linked thrombocytopaenia with thalassaemia
CEP: Congenital erythropoietic porphyria
TMD: Transient myeloproliferative disorder
AMKL: Acute megakaryoblastic leukaemia
PGT-HLA: Preimplantation genetic testing-human leukocyte antigen
Hb: Haemoglobin
PCR: Polymerase chain reaction
MALBAC: Multiple annealing and looping-based amplification cycles
WGA: Whole genome amplification
ADO: Allele drop-out
SNP: Single nucleotide polymorphism
CNV: Copy number variation
FET: Frozen embryo transfer
CMA: Chromosomal microarray analysis
ACMG/AMP: American College of Medical Genetics and Genomics and the Association for Molecular Pathology
